# Modelling Osteoporosis in Pre-Clinical Research—Challenges, Trends and New Approaches

**DOI:** 10.3390/cells14211649

**Published:** 2025-10-22

**Authors:** Johannes Plank, Alexandra Damerau, Madison Skye Chacon, Paula Hoff, Frank Buttgereit, Moritz Pfeiffenberger

**Affiliations:** 1 Department of Rheumatology and Clinical Immunology, Charité-Universitätsmedizin Berlin, Corporate Member of Freie Universität Berlin, Humboldt-Universität zu Berlin, 10117 Berlin, Germany; johannes.plank@charite.de (J.P.); paula.hoff@charite.de (P.H.);; 2 German Rheumatism Research Centre (DRFZ) Berlin, a Leibniz Institute, 10117 Berlin, Germany; 3 Endokrinologikum Berlin, Medizinisches Versorgungszentrum (MVZ) am Gendarmenmarkt, 10117 Berlin, Germany

**Keywords:** osteoporosis, in vivo models, in vitro models

## Abstract

**Highlights:**

**What are the main findings?**

- A comprehensive comparative analysis of current approaches to model osteoporosis, including in vivo, in vitro, and in silico systems.
- Identification of key limitations of current animal models and the emerging potential of advanced human-derived in vitro platforms.

**What are the implications of the main findings?**

- Advanced in vitro and organ-on-a-chip technologies are useful tools for bridging the translational gap between experimental research and clinical application.
- Integrating human-based and computational models may accelerate the development of more predictive, mechanism-driven therapeutic strategies.

**Abstract:**

Osteoporosis is a bone disease characterized by low bone mass and changes in bone architecture, often leading to fractures and thereby decreased functional status in affected patients. About 200 million people worldwide suffer from osteoporosis, with women being affected earlier in life and more often than men. Various factors, such as genetic background, comorbidities, alcohol abuse, and medications such as glucocorticoids, are known to contribute to the development of osteoporosis. Due to the changing demographics, osteoporosis is becoming increasingly prevalent, and with this, the rate of fractures is expected to increase in the coming years. To investigate therapeutic options for treatment and to elucidate disease-causing mechanisms, various in vivo and in vitro osteoporosis models have been developed. In vivo models, in particular small animal models, remain the gold standard for osteoporosis research and the most used model to illustrate osteoporosis is the ovariectomized mouse. While in vivo models largely reflect the systemic and biological conditions, the transferability of findings to human patients is low and ethical concerns for laboratory animals must be considered. Thanks to tremendous technological improvements, such as on-a-chip platforms and high-end bioreactor systems, sophisticated in vitro models are of growing interest. These models offer the possibility of using complex cell systems, human cells from single donors, and 3D models, thus bridging the transferability gap, providing a platform for the introduction of personalized precision medicine, and ultimately replacing animal testing. Here, we summarize and discuss recent in vivo, in vitro, and in silico osteoporosis research approaches.

## 1. Introduction

Osteoporosis is the most prominent bone loss disease, leading to bone fragility and fractures. Due to the aging population, osteoporosis is becoming increasingly prevalent, as illustrated by the rise in hip fractures from 1.3 million per year in 1990 to 2.7 million in 2010 [1,2]. Globally, around 500 million people over age 50 are affected by osteoporosis, leading to about 37 million fragility fractures each year (≈70 per minute). In Europe alone, an estimated 32 million individuals aged 50+ have osteoporosis, with women disproportionately affected [3].

The World Health Organization defines osteoporosis as having a bone mineral density (BMD) of less than 2.5 standard deviations below the average of healthy, young women, i.e., a T-score of ≤−2.5 SD. A commonly used method to assess bone density is dual-energy X-ray absorptiometry (DXA) [4]. Osteoporosis, characterized by a change in bone microarchitecture and an increased risk of fracture [5,6], can be assessed more precisely with the trabecular bone score (TBS), a gray-scale analysis of DXA images [7]. A considerable fraction of patients with no pathological BMD had TBSs that served as indications to start antiresorptive therapy [8].

In a healthy state, bone tissue consists of around 60% inorganic substances, 8–10% water, and 30% organic substances. The inorganic phase is composed mainly of hydroxyapatite, a form of calcium phosphate, while the organic substance consists mainly of collagen I (around 98%) and many proteins such as osteonectin, osteopontin, osteocalcin, and bone sialoprotein. Most bones generally consist of an outer, harder, more compact bone structure called the compacta and the inner trabecular structure, which contains bone marrow and fat cells, called the cancellous bone. The most prominent cell types inside bone are bone-forming osteoblasts, mechanosensitive osteocytes, and bone-resorbing osteoclasts [9]. Osteocytes are derived from osteoblasts after they have ceased matrix formation. They are involved in bone homeostasis and remodeling, and influence bone resorption through the secretion of sclerostin, a protein that induces osteoclasts. They also secrete fibroblast growth factor 23, an endocrine factor that regulates phosphate metabolism [10]. In addition, osteocytes play an important role in mechanosensing, detecting mechanical stress, and translating these signals into biochemical signals that regulate bone remodeling. Using their extensive network of dendritic processes, they communicate with other bone cells to coordinate the deposition and resorption of bone tissue in response to mechanical stress [11]. Whereas osteoblasts and osteocytes derive from the mesenchymal lineage, osteoclasts are of hematopoietic origin.

**Figure 1:**
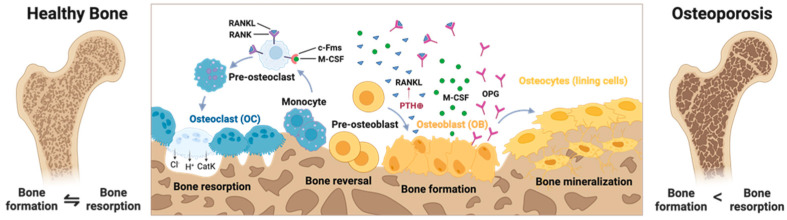
On the left is a cross-section of a healthy bone in which bone formation and resorption are in balance so that bone density and strength are maintained. The middle panel shows the dynamic process of bone remodeling, which consists of four key phases: Bone resorption: pre-osteoclasts (blue cells) differentiate into active, multinucleated osteoclasts under the influence of signaling molecules such as RANKL and M-CSF. Osteoclasts break down bone by degrading the mineralized matrix, releasing calcium and phosphate. Monocytes and other cells clean the resorbed bone surface to prepare it for the deposition of new bone by osteoblasts. Osteoblasts (yellow cells) synthesize and deposit new bone matrix. PTH stimulates bone remodeling, while osteoprotegerin (OPG), a decoy receptor secreted by osteoblasts, binds RANKL to inhibit excessive osteoclast activity. The deposited matrix is mineralized over time to form strong, mature bone. Osteocytes (lining cells) derived from osteoblasts embedded in the matrix regulate mineralization and respond to mechanical stress. The right section highlights the imbalance in bone remodeling in primary or secondary osteoporosis, where bone resorption exceeds bone formation. This leads to lower bone density, a weakened structure, and a higher risk of fracture. In summary, the balance between bone resorption and formation is critical to maintaining healthy bone. Disturbances in signaling pathways, such as excessive RANKL or decreased OPG, promote the progression of osteoporosis. Created with BioRender.com.

Bone homeostasis in healthy bones is an accurately adjusted equilibrium between the degradation of osseous substance by osteoclasts, the formation of new bone by osteoblasts, and the following mineralization process orchestrated by osteocytes (Figure 1). This process is mediated by the interaction between receptor activator of nuclear factor κB (RANK) on osteoclast precursors and its ligand RANKL, which is expressed by osteoblasts and stromal cells. Osteoblasts also secrete macrophage colony-stimulating factor (M-CSF), which, together with RANKL, recruits osteoclast precursors and promotes their differentiation and maturation into functional osteoclasts, thereby regulating bone (Figure 1). The bone remodeling process contributes to stability by helping bones adapt to mechanical load and regulates serum calcium levels. Resorption of bone is mediated by Ca^2+^ sensors in the parathyroid gland by secretion of parathyroid hormone (PTH) if Ca^2+^ levels are low. Subsequently, osteoblasts respond by secreting RANKL, triggering osteoclasts to secrete protons and activate H^+^-ATPase, which breaks down hydroxyapatite. To resorb the organic substance, osteoclasts secrete proteases such as cathepsin K, degrading the extracellular matrix. Furthermore, bone resorption can be enhanced by elevated levels of activated vitamin D_3_, which promotes calcium absorption and regulates calcium homeostasis. Conversely, osteoblasts secrete osteoprotegerin (OPG) in response to calcitonin, which acts as a decoy receptor for RANKL, thereby inhibiting the differentiation and activity of osteoclasts and reducing bone resorption [12]. In summary, PTH and activated vitamin D3 are released when serum calcium levels are low and initiate the breakdown of bone, while calcitonin stops this process when serum calcium levels increase. The following section describes the pathological processes if this equilibrium is disturbed [9].

Several mechanisms play into the development of osteoporosis and influence the balance between bone resorption and bone formation.

Osteoporosis is differentiated into primary and secondary osteoporosis based on factors that trigger the disease. Primary osteoporosis is caused by aging and menopause without having any accompanying disease [13]. Secondary osteoporosis is caused by a concomitant disease and medications that promote osteoporosis as recently reviewed by Sobh et al. and Mirza & Canalis [14,15] (Table 1).

Primary osteoporosis is caused by age and a sex hormone deficiency, although the precise influence of the individual factors remains unclear. However, the combination of different negative predispositions including the genetic background can lead to the development of osteoporosis. A widely known risk factor is the onset of the menopause, which is accompanied by a marked reduction in hormone levels. After menopause, the level of sex hormones, mainly estrogen, produced by the ovaries, is substantially decreased, leading to increased osteoclast activation. Furthermore, inflammatory cytokines like TNF (RANKL is a member of this superfamily), IL-1, and IL-6 increase, enhancing bone resorption and leading to postmenopausal osteoporosis. These cytokines are suppressed in premenopausal women. Moreover, estrogen stimulates the expression of osteoprotegerin, a decoy receptor for RANKL [16]. While there is no known equivalent to menopause in males, the serum levels of testosterone and estradiol decrease with age due to increasing levels of sex hormone binding globulin (SHBG) and growing gonadal insufficiency, which can result in osteoporotic changes similar to those seen in postmenopausal women [17].

Age-related bone loss begins in the third decade of life after the peak bone mass has been reached. There are various factors contributing to impaired bone metabolism with age, including reactive oxygen species (ROS) that arise from internal factors, such as respiration, and external factors like ionizing radiation. Aging is correlated with increasing levels of ROS [16,18]. The lack of growth hormone (GH) and insulin-like growth factor 1 (IGF-1), which are less secreted with age and lead to decreased bone metabolism, can also serve as an explanation for age-related bone loss. Furthermore, low levels of IGF-1 are associated with an increased risk of fractures, as shown in the OFELY study [19,20].

The most common form of secondary osteoporosis is glucocorticoid-induced osteoporosis (GIOP) [21,22]. About 2.5% of the patients aged 70–79 are frequently prescribed glucocorticoids (GCs), enhancing the risk for fractures, especially in the femoral neck and vertebrae [23]. The risk for fractures increases rapidly after the beginning of GC therapy and is strongly dose-correlated [24]. Additionally, there is an association between increased incidence of osteoporosis and patients taking GCs for more than three years [25]. Although physiological concentrations of GCs are required for osteoblast differentiation, higher concentrations inhibit differentiation and increase apoptosis. In addition, osteoblast function is inhibited by GCs, resulting in reduced expression of collagen I. Furthermore, the disruption of bone formation is accompanied by direct and indirect effects on osteoclasts, leading to increased expression of RANKL and M-CSF, which in turn results in enhanced osteoclastogenesis, a longer lifespan, and increased activity of the osteoclasts [26]. All inflammatory diseases contribute to an increase in cytokine-mediated bone resorption [21,23]. However, studies have shown that the impact of glucocorticoids may be dose dependent. Wiebe et al. concluded that GC doses of ≤5 mg/day prednisone equivalent may not be associated with negative effects on bone mineral density in a cohort of inflammatory rheumatic and musculoskeletal diseases [27]. Additionally, studies performed by Palmowski et al. show no association between current or cumulative GC intake and impaired bone density in patients suffering from polymyalgia rheumatica, giant cell arteritis, and other vasculitides [28]. Another study by Adami et al. investigated the effects of GCs on bone state in patients with inflammatory rheumatic musculoskeletal disorders. They show that GCs with doses below 2.5 mg/d did not affect bone mineral density when treated with antiresorptive drugs. This was not seen in doses over 5 mg/d; however, it should be noted that the study was not sufficiently adjusted, nor was disease activity taken into account [29].

Another form of secondary osteoporosis in humans is disuse osteoporosis, which is when immobilization causes bone loss affecting the microarchitecture of the bone in a similar way to postmenopausal osteoporosis. Frequently occurring reasons for disuse osteoporosis are spinal cord injuries and neurological disorders affecting activity and physiological loading on bone, like encephalomyelitis disseminata. Other causes of bone loss due to inactivity are extensive bed rest and space travel. Despite systemic bone loss, local immobilization, e.g., after a fracture, can cause local disuse osteoporosis [30].

In the context of the increase in fractures associated with fragility, several new drugs have been developed that allow a more targeted treatment of osteoporosis. Frequently used are antiresorptive drugs like bisphosphonates and denosumab (an antibody against receptor agonist of NF-κB ligand (RANKL)), and the osteo-anabolic agents teriparatide and abaloparatide; romosozumab (an antibody targeting sclerostin) exhibits a dual osteo-anabolic but also antiresorptive mode of action [5,31]. There is also a large number of substances that may act in an osteo-protective way, as indicated in in vitro studies for instance as Hansen et al. showed for tofacitinib [32]. Gaber et al. studied the impact of tofacitinib on fracture healing. They showed that MSCs were not affected by therapeutic doses. Beyond this, differentiation towards osteoblasts is promoted, while the activity and differentiation of osteoclasts are decreased. It was concluded that tofacitinib has a positive effect on the treatment of bone erosions in rheumatoid arthritis [33]. This indicates that various approaches need to be developed to address the clinical needs in treating osteoporosis and improving the management of related diseases, particularly given the significant mortality rate following an osteoporotic fracture. [34]. To gain deeper insight into the mechanisms behind osteoporosis, various in vivo and in vitro approaches have been developed. However, the results vary depending on the model chosen and must therefore be selected carefully, according to the relevant question.

The German Dachverband Osteologie e.V. recommends all patients suffering from osteoporosis to take about 1000 mg of calcium with food and between 800 and 2000–4000 units of vitamin D, even without indications for anti-osteoporotic therapy. Recent research has shown that a high single dose of vitamin D (300,000–500,000 units) increase the risk of fractures and falls. Despite this, serum levels of activated vitamin D should not exceed 250 nmol/L to prevent toxicity. Supplementation is only necessary if the alimentary intake is not ample. Additional antiresorptive or osteo-anabolic medication is needed if there is an enhanced risk for further fractures. The current German guideline for the treatment of osteoporosis no longer gives a T-score above which therapy should be started; only the absolute fracture risk is used as an indication. Factors for an increased risk are, e.g., a T-score below 2.5 or a therapy with glucocorticoids like prednisolone for more than three months [35]. Studies performed by Loddenkemper et al. showed that there is a negative correlation between bone density of the femoral neck and bone resorption parameters N-terminal telopeptide and pyridinoline [36]. Men receive the same therapy as women, though the therapy is less effective in men. Five common classes of anti-osteoporotic drugs are listed below (Table 2).

Keeping the rudimentary understanding of osteoporosis and its treatment in mind, further research is needed to fill this gap. There are several in vitro and in vivo models that can help elucidate osteoporosis and its treatment, which we present in the following.

## 2. Modeling Osteoporosis In Vivo

Fundamentally, an in vivo model should be well planned under stringent conditions. This includes planning the costs and the number of animals used based on statistically relevant considerations, observing basic ethical principles, and including biologically relevant characteristics [37].

In addition, the transferability of the collected data to the human system and the associated clinical relevance play an increasingly important role. Furthermore, the species used in the study should be discussed at the planning stage and a decision made considering the points mentioned above [37]. To date, small animal models such as mice and rats, and occasionally hamsters, fish, and guinea pigs have been used to model osteoporosis. Large animal models include dogs, sheep, and cats, although experiments on ferrets and non-human primates are also used [38]. These animal models can be roughly divided into three categories, based on the mechanism by which osteoporosis develops: (i) post-menopausal osteoporosis, (ii) disuse osteoporosis, and (iii) glucocorticoid-induced secondary osteoporosis. To generate postmenopausal osteoporosis, the test animals are subjected to an ovariectomy, which leads to increased bone resorption and a slightly reduced ability to build bone due to hormonal factors. Mice, rats, and sheep are the main animals used for this purpose, while non-human primates are used in follow-up studies [14].

Mice, rats, and dogs are also usually used to simulate disuse osteoporosis. For experimental purposes, the animal’s hind limb is immobilized, which leads to non-load-bearing conditions and thus the development of osteoporotic changes. Increased bone resorption is also achieved in these models along with a significant reduction in bone formation. To induce GIOP, glucocorticoids are administered to the test animals via the drinking water or by repeated injection or infusion or via pellet implantation [39]. These also lead to increased bone resorption and greatly reduced bone formation. Mice, rats, sheep, rabbits, and dogs are mainly used for this purpose. In addition to these physically- or chemically induced animal models, a variety of transgenic mouse models exist to simulate osteoporosis in vivo [40,41] (Table 3).

In animal models of osteoporosis, the diagnosis of the disease is confirmed by a combination of various diagnostic methods. The BMD is often determined using DXA or peripheral quantitative computed tomography (pQCT), which measure bone mass or density, comparable to the clinical T-score in humans. Typically, these measurements are taken at the beginning of the experiment and between four and twelve weeks after ovariectomy or hormonal treatment. Bone microarchitecture is analyzed using µCT to quantify trabecular and cortical parameters such as bone volume/BV, trabecular thickness (Tb.Th), trabecular number (Tb.N), and trabecular spacing (Tb.Sp). This method is considered the gold standard for confirming typical trabecular deterioration in osteoporosis. In addition, bone histomorphometry is used, either static or dynamic, for example by TRAP staining or calcein double labeling, to determine the number and activity of osteoclasts and the rate of bone formation, thereby demonstrating an increased bone turnover rate and structural loss. Biomechanical testing using three-point bending, compression, or nanoindentation serves to evaluate the mechanical strength, stiffness, and fragility of bone and to demonstrate the functional consequences of bone loss. Biochemical markers of bone metabolism, such as TRAP5b and CTX-I for resorption or P1NP and osteocalcin for formation, can be used to detect systemic changes in bone metabolism at an early stage, often as early as one to two weeks after ovariectomy or drug intervention. Together, these methods enable comprehensive, multimodal diagnosis of osteoporosis [45,46,47]. Moreover, the FDA guidance provides recommendations for nonclinical bone quality studies that support the development of osteoporosis drugs. Studies should include two doses, reflect the intended clinical dosing regimen, and be of sufficient duration to cover multiple bone turnover cycles. Assessments must include bone turnover markers, bone mass and density (DXA, pQCT), microarchitecture (µCT, histomorphometry), and biomechanical strength, considering correlations between density and strength parameters. For biologics, a single pharmacologically relevant species or a transgenic model may be acceptable if species-specific receptor interactions limit standard tests. However, these are not fixed regulations for detecting osteoporosis in laboratory animals, but rather non-binding recommendations [48].

### 2.1. In Vivo Models of Post-Menopausal Osteoporosis

Postmenopausal osteoporosis is a bone formation disorder caused by estrogen deficiency. The main methods used for the simulation of postmenopausal osteoporosis are ovariectomy in mice and rats, hormonal manipulation, or the use of naturally aging animals. These model systems offer various advantages but also limitations, depending on the research question [49]. Removal of the ovaries results in a rapid drop in estrogen levels that mimics the menopausal state. This is a well-established and widely used method in osteoporosis research, with many established protocols for the analysis of bone parameters. However, the operation itself requires a high level of expertise from the surgeon and causes postoperative stress in the animals used [50].

Postmenopausal osteoporosis can also be simulated in vivo by manipulating hormonal levels through chemical induction, for example by GnRH analogs [51]. Additionally, osteoporosis can also be caused by specific knock-out variants (Esr1 mice) in animal models [52]. However, these genetically engineered models often come with undesirable side effects.

Another approach is to use naturally aging animals, such as rodents and zebrafish. With these models, it is possible to analyze osteoporotic changes without surgical intervention, but they require a long period to collect data, and osteoporosis does not occur spontaneously in every animal [53].

The experimental achievement of an osteoporosis-like bone structure varies between species. While in mice it is usually established 4–8 weeks post-OVX in many study designs, this process takes more than 12 weeks in the rat model. Naturally, the effect also depends on the skeletal structure and age of the animals used. Manipulating hormonal levels will take a little longer [54,55] (Table 4).

In recent years, these models have been used to uncover intriguing processes in the progression of osteoporosis and to develop new therapeutic strategies. In recent years, this topic has been the focus of multiple studies. Deficiency of the sex hormone estrogen promotes the differentiation and maturation of osteoclasts by activating T lymphocytes [56]. Using an in vivo rat model, Shao et al. demonstrated that estrogen promotes osteoclast apoptosis by down-regulating microRNA-181a, which normally suppresses NF-κB, leading to the accumulation of Fas ligand and subsequent induction of osteoclast cell death [57]. Another study showed that miR-181a exerts a negative regulatory effect on CD4+T lymphocyte apoptosis induced by bone marrow-derived mesenchymal stromal cells [56]. Zhi et al. also demonstrated the importance of the NF-κB and MAPK signaling pathway. Using l-tetrahydropalmatine, a plant alkaloid that is considered a promising agent for various osteoclastogenesis-related diseases, they were able to show a clear improvement of disease-related characteristics in ovariectomized mice. They were able to show that l-tetrahydropalmatine blocks the interactions between RANK and TRAF6, inhibiting the NF-κB and MAPK signaling pathways, and thereby inhibiting osteoclastogenesis [58].

Sirtuin (SIRT1) is another promising agent for the treatment of osteoporotic bone changes. To model postmenopausal osteoporosis, Zainabadi et al. used ovariectomized female mice. Using KO mice models, they were able to show that SIRT^-^ mice had lower bone mass. In addition, it was shown that a specific SIRT1 agonist led to an improvement in bone growth. Thus, indicating that SIRT1 is a positive regulator or enhancer of bone mass and therefore represents a promising new therapeutic target [59]. In a study with rats, Miao et al. showed a protective effect of genistein on bone. Genistein is an isoflavone from soybeans and has similar effects as estrogen (phytoestrogen). The phenomenon of bone-protective properties has been known, but Miao et al. showed the dependence of the parathyroid hormone-parathyroid hormone receptor axis on genistein’s effects. Therefore, genistein could be another potential option for the prevention and treatment of postmenopausal osteoporosis, as it has already been shown to be well tolerated with few side effects and the clinical benefits are strongly suspected [60]. Similarly, when the recently developed compound alendronate-CGS21680 conjugate (MRS7216) was administered to C57BL/6J wild-type mice with established osteoporosis in a study by Larrañaga-Vera et al., there was less loss of bone mass and increased new bone formation. This effect was even stronger than treatment with a common alendronate alone, possibly indicating a synergistic effect of the conjugate [61].

In another approach, some efforts do not intervene directly in osteoclastogenesis but rather focus on the growth of new bone. In a study by Gera et al., they investigated the therapeutic potential of the flavonoid naringenin. For this purpose, ovariectomized rats were administered a nanosuspension of naringenin. After therapy, the level of important parameters in the serum (calcium, phosphorus, acid phosphatase, alkaline phosphatase, osteocalcin) recovered, and increased bone homeostasis was achieved [62].

Many studies also use ovariectomized sheep as a model, often in combination with diet deficiency (e.g., Vitamin D, calcium) and/or GC administration. This is primarily because sheep are easy to keep and handle, cost less compared to other large animals and are accepted in society as research animals. Sheep models are used to study: the response to new therapies for postmenopausal osteoporosis; the effects of mechanical stimulation particularly in the low frequency range; the tolerability and applicability of orthopedic implants in the human system; and the use of bioactive ceramics to strengthen the vertebral bodies; or in the field of dental medicine [63]. Furthermore, these models, as well as ovariectomized goats, are used in studies aiming to improve the healing process in fragility fractures or to improve the repair of bone defects in osteoporotic bones [64]. Regarding this topic, a study by Eschler et al. on an ovariectomy sheep model showed that cementless titanium mesh fixation was successful without intraoperative complications. In all cases, bony consolidation and good results in biomechanical tests were demonstrated. Micro-CT analysis demonstrated stable integration of the scaffold into the bone and bony fracture healing. The product is now being used clinically [65]. Studies by Dittmer et al. were able to show that the protein Klotho, known as an anti-aging hormone, correlated with increased bone density in sheep. From this, they concluded that Klotho could play a key role in combating osteoporosis; however, further studies are needed to resolve the signal cascade and the underlying pathway [66]. Interestingly, the metaphyseal defect model is mostly used to study adequate fracture healing in sheep. In a dual-purpose study, Rupp et al. used ovariectomized sheep in conjunction with the administration of methylprednisolone and a deficiency diet. By injecting a special fluorochrome, it was possible to analyze various bone parameters over time. This allows osteoporotic changes as well fracture healing to be monitored simultaneously in the same animal and is therefore compatible with the 3R principle [67].

Non-human primates such as macaques or other old-world monkeys are, in principle, an excellent model for osteoporosis in humans. This is mainly because their reproductive endocrine system is very similar to that of humans, and it has a comparable influence on bone metabolism. With increasing age, the monkeys showed a disturbance of bone homeostasis and an increased skeletal turnover very similar to that of humans, which can be explained by the reduced estrogen secretion during menopause. In addition, the osteonal remodeling of the bone cortex in the Haversian system is very similar to that of humans. For this reason, these animals have often been used in studies on osteoporosis, although their use is strictly regulated due to ethical issues [68,69,70]. Remarkably, Saltzman et al. revealed that common marmoset monkeys did not lose any bone mass after estrogen depletion, suggesting this species may possess unique adaptations to avoid bone loss related to estrogen depletion. However, the factors involved in this protective effect are still elusive and need to be further investigated [71]. Currently, non-human primate studies regarding osteoporosis are limited to studying the material used in implants for bone fractures, such as testing the safety and osseointegration of novel titanium screws [72].

Interestingly, although the positive effect of deer bone extract on bone homeostasis has been known, more recent studies have focused on the effects on ovariectomized mice. Various studies have shown a positive effect on bone formation or osteoporosis-related symptoms, which could lead to an improved therapeutic strategy [73,74].

### 2.2. In Vivo Models of Disuse Osteoporosis

Another frequently used model to simulate osteoporosis in vivo is the so-called disuse osteoporosis model, which is often the result of a lack of exercise, training, or regular weight bearing. For this purpose, the hind leg or tail is usually immobilized so that no loading is exerted on the bone inducing osteoporotic changes. In this case, mice, rats, and dogs are commonly used. Another method is the immobilization using casts. The duration of disuse to which the animals were exposed for the development of osteoporosis ranged from 2 weeks in mice and rats to 1 year in dogs, with an average of 3 weeks. In general, larger animals such as dogs, monkeys, rabbits, and sheep are exposed to longer periods of non-use than smaller animals such as rodents [75] (Table 5).

Recently, these models to mimic disuse osteoporosis have been employed to shed light on essential processes. It has long been known that the hypoxia inducible factor 1 subunit alpha (HIF-1α) regulates bone formation after osteogenic mechanical stress [76] and promotes appropriate fracture healing [77]. The study by Bie et al. used the tail-suspended mouse model to demonstrate that HIF-1α/c-Myc signaling may also be required for osteoclasts, thus mediating an important role in cell metabolism in the development of disuse-induced osteoporosis. Among others, it was shown that genetic deletion of HIF-1α in osteoclasts improved bone resorption in bone. On the other hand, pharmacologic treatment with a HIF-1α inhibitor resulted in protection of trabecular and cortical bone from disuse-induced osteoporosis [78]. The development of osteoporosis is often credited to a lack of exercise. However, a study by Lyu et al. found preliminary evidence that zinc has a protective causal relationship with osteoporosis in non-users by promoting exercise and immunity [79]. Extracellular vesicles (EVs) generally serve as important messengers in cell communication and are found in fluids such as blood, urine, and saliva. A study by Capariello et al. showed that EVs also play a role in osteoporosis. Using a disuse mouse model, they identified molecular signatures in EVs that indicate their potential as tools for the diagnosis and monitoring of osteoporosis clinically [80]. In a study by Xiao et al., the role of exosomes in bone homeostasis was confirmed in a disuse mouse model and in vitro. Exosomes were obtained from mechanically stretched bone-marrow-derived mesenchymal stem cells by ultracentrifugation. These prevented the formation of osteoclasts in the model by suppressing the RANKL-induced NF-κB signaling pathway. Therefore, these exosomes could possibly be used in the future as a potential therapeutic option against abnormal bone loss [81]. There is increasing evidence that microRNAs (miRNAs) play an essential role in mechanosensing to regulate osteogenesis. However, no mechanosensitive miRNAs have yet been identified in human bone samples. In a study by Chen et al., a microRNA (miR-138-5p) was identified for the first time as a mechanosensitive factor in human bone. Its occurrence correlated strikingly with reduced bone formation, and inhibition of MiR-138-5p led to an increased anabolic response in the mouse model, which counteracted the reduced differentiation into osteoblasts. Inhibition of this specific miRNA could therefore represent an interesting therapeutic option in the treatment of osteoporosis [82]. Furthermore, a study by Hu et al. showed that the targeted silencing of the expression of miRNA-132-3p could counteract the development of osteopenia. This was particularly evident by promoting osteogenic differentiation of mesenchymal stem cells and osteogenesis in the mouse model [83].

### 2.3. In Vivo Models of Glucorticoid-Induced Osteoporosis

The most common form of secondary osteoporosis is glucocorticoid-induced osteoporosis (GIOP) [14]. Most animal model systems use small animals such as mice, rats, and rabbits. Moreover, large animal models consist mostly of dogs and sheep [44]. In mouse models, glucocorticoid-induced osteoporosis is typically induced within three weeks, with prednisolone administered at doses ranging from 0.8 to 4 mg/kg per day. In rat models, the duration of treatment varies significantly depending on the substance and dose: dexamethasone is usually administered over 6 to 8 weeks (0.1–25 mg/kg), while prednisone is given for up to 90 days at lower doses (1.5 mg/kg/day) and in long-term studies even for over 90 weeks (6 mg/kg/day). In large animal models, a significantly longer period of time is required for osteoporotic changes to develop: in rabbits, dexamethasone is administered over 12 weeks and in sheep, prednisolone is administered over seven months (0.6 mg/kg, five times a week) [47].

A study by Ge et al. investigated the protective effect of naringin, a bitter substance known primarily as a natural ingredient of grapefruit, against GIOP in vitro as well as in a GIOP-induced rat model in vivo. They showed in vitro that naringin increased the proliferation and differentiation of osteoblasts. In addition, autophagy was increased. In the rat model, the administration of naringin primarily improved bone density. In contrast, administration of the PI3K/AKT/mTOR signaling pathway inhibitor LY294002 reversed this effect. Their results suggest that naringin could be a factor in the protection and preservation of bone tissue through increased osteoblast proliferation and enhanced autophagy [84]. Regarding the PI3K/AKT/mTOR signaling pathway, the group of Li et al. revealed that melatonin inhibited ferroptosis by activating the PI3K/AKT/mTOR signaling pathway, reducing the occurrence of GIOP [85]. Another active compound that could be a promising product for the treatment of osteoporosis is tocotrienol, which protects against oxidative damage, reduces lipid peroxidation, activates of the Wnt/β-catenin signaling pathway, and lowers the RANKL/OPG ratio. Several preclinical studies have already shown its positive effect in preventing bone loss in in vivo models. In addition, administration of tocotrienol increased osteogenic differentiation, improved structural quality and integrity of bone, and integral regeneration of bone tissue. However, the therapeutic benefits of the compounds have not been confirmed in clinical trials [86].

### 2.4. Limitations

Although mice and rats are frequently used, they are not an ideal model for the representation of human osteoporosis for several reasons. Human cortical bone undergoes continuous remodeling throughout life. In contrast, the cortical bone of mice and rats rarely undergoes Haversian remodeling and therefore generally does not contain osteons [68]. In particular, the selection and equally important the interpretation of large animal model data for osteoporosis research depend on understanding the differences between humans and quadrupeds in bone properties. Rapid bone loss after ovariectomy is mostly consistent with human conditions. However, FDA guidelines still recommend studying long bones and vertebrae in models of postmenopausal bone loss. The presence of bone remodeling to study osteoporotic changes is of tremendous importance, as it reflects an important characteristic of human bone. In addition, humans have proportionally more lamellar bone, whereas large mammals have more plexiform bone with unique mechanical properties. The mechanical load and thus the effect of mechanical forces on the skeletal system differ between quadrupeds and humans due to the upright or quadrupedal gait and the morphology of the vertebral bodies. The bone loss caused by ovariectomy is often assessed by biopsies of the iliac crest. Although this is technically easier to visualize in large animal models compared to mice and rats, there are concerns regarding comparability and relevance compared to other affected regions, such as the femoral neck or other vertebral bodies. Terminology studies at key weight-bearing sites provide a more direct assessment of osteoporosis-related changes, considering density and micro-architectural changes in typical fracture-prone regions [87,88]. Of note, ethical considerations and ethical justifiability should also be considered, as many test series involve pain and suffering for the animals used.

## 3. Modeling Osteoporosis In Vitro

**Figure 2:**
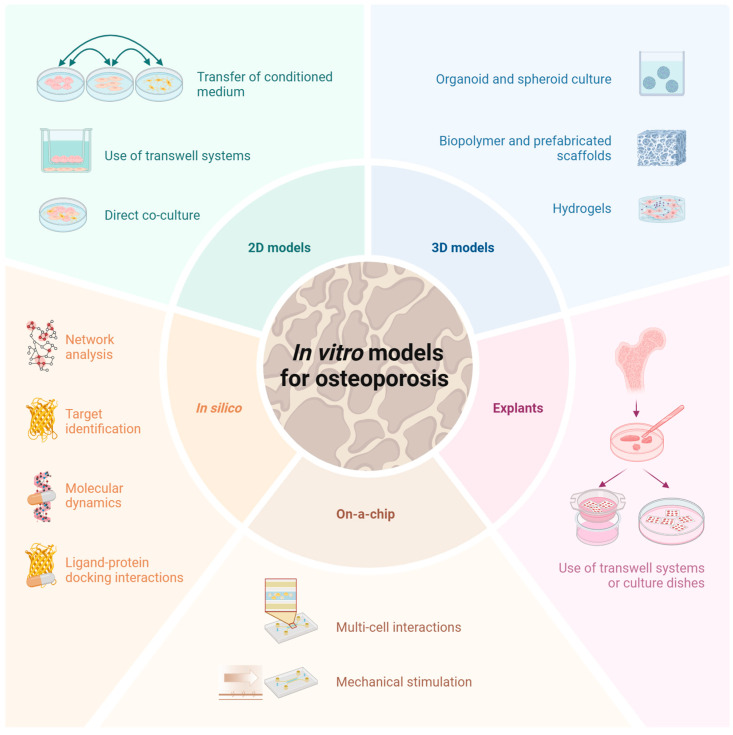
Schematic overview of different in vitro approaches to study osteoporosis and its pathophysiology. The models presented are divided into five main categories: 2D models, 3D models, in silico models, on-a-chip approaches, and explants. Each category represents different experimental approaches to simulate the complex cell and tissue interactions which are critical to understand and develop therapeutics for osteoporosis research. This illustrates that a multidisciplinary approach is required to adequately study osteoporosis. Thus, each category offers specific advantages and applications, and underlines the importance of the appropriate model depending on the research question and experimental requirements. Created with BioRender.com.

As in vivo models of osteoporosis often lack transferability, human-derived in vitro bone models can correct for this issue. There are different approaches for establishing in vitro bone models, especially for osteoporosis. Here, we focus on summarizing current articles on bone models and osteoporosis (summarized in Figure 2).

### 3.1. Two-Dimensional In Vitro Models

The first models to resemble the microenvironment of the bone are 2D models, and they have been widely used as drug testing platforms [89,90,91]. Two-Dimensional models are based on monolayer cell cultures and are easy to handle and offer a high degree of control over cell and environmental conditions. Approaches include the transfer of conditioned medium, which allows the study of cell–cell interactions by transferring the medium generated by a cell culture to other cells. The use of transwell systems allows the investigation of paracrine signaling pathways between cells that are spatially separated but connected by a semipermeable membrane. Another approach is the use of direct co-culture setups, where different cell types are cultured together in order to study direct cell–cell interactions, such as between osteoblasts and osteoclasts or their respective precursors. A common use for 2D in vitro models is pre-testing for animal models [89,92]. Though, 2D models fail to imitate certain physiological conditions like developing a 3D shape of the skeleton [90].

Zhu et al. co-cultivated human SaOS-2 (an osteosarcoma cell line) with human leukemia cells (THP-1) to obtain a functional system of osteoblasts and osteoclasts. The activity of osteoclasts was determined by measuring the activity of carbonic anhydrase and tartrate resistant acid phosphatase, both enzymes expressed for resorbing bone extracellular matrix. The ability of osteoblasts to deposit calcium phosphate was assessed using the alizarin red assay. Gene expression, protein levels, and total DNA were evaluated as well. Further on, osteoporosis was induced using cigarette smoke extracts (CSE). Different concentrations of CSE were tested, overall showing an increase in osteoclast activity and a decrease in matrix deposition [93]. In another study, Cha et al. tested bisphosphonates (alendronate, ibandronate, and zoledronate) to evaluate their effect on patient-derived mesenchymal stromal cells. Therefore, they used bone marrow from healthy subjects and patients with osteoporosis. After exposure to the therapeutics for 24 hr, alkaline phosphatase activity assay, alizarin red staining, calcium assay, and qPCR were performed. They found that bisphosphonates have a higher effect on osteogenesis in cells derived from osteoporotic bones than from healthy ones, suggesting that bisphosphonates are more effective in the treatment rather than in the prevention of osteoporosis [94].

A study performed by Hu et al. showed that the micro-RNA miR-491-3p is upregulated in postmenopausal osteoporosis unlike cathepsin s, which is downregulated. To evaluate this, the human osteoblast cell line hFOB1.19 was used, and an overexpression of miR-419-3p and cathepsin s was achieved by transfecting miR-491-3p mimic and pcDNA3.1-CTSS in the cell line. They found that the overexpression of miR-491-3p promotes the proliferation and differentiation of osteoblasts and inhibits apoptosis. Furthermore, they observed a negative regulation of cathepsin s by miR-491-3p [95].

In a study performed by Lv et al., they investigated the function of the miR-27b/PPARγ, an inhibitor of osteoblast formation, axis in glucocorticoid treated osteoblasts. A model of glucocorticoid-induced osteoporosis was set up using mouse-derived MC3T3-E1 pre-osteoblast cells. After the cells were differentiated into osteoblasts, osteoporosis was induced using 1 µM dexamethasone. They performed an overexpression and knockdown of miR-27b-3p and silenced PPARγ2, therefore limiting adipogenesis. Key findings of the study are that miR-27b-3p attenuates the effects of dexamethasone on osteoblasts and that PPARγ2 can abrogate the effects of miR-27b-3p [96].

In an alternative approach, instead of determining the decreased activity of osteoblasts, one can identify substances leading to a higher activation of osteoclasts. This approach was pursued by Brom et al., when they evaluated the effect of immune checkpoint inhibitors on osteoclast function. Immune checkpoint inhibitors are widely used in treatment of cancer and have a wide range of potential applications [97]. A study from Brom et al. showed that certain immune checkpoint molecules can modulate bone resorption, with PD-1, PD-L1, GITR, and CD47 promoting osteoclast formation, while PD-L2, DPPA-1, TIGIT, and Tim-3 inhibit it. These effects could be neutralized by the respective antibodies [98]. Taken together, 2D in vitro bone models provide valuable, controllable platforms for studying osteoblast–osteoclast interactions and drug effects, but they remain limited in mimicking the complex three-dimensional and physiological environment of bone tissue.

### 3.2. Three-Dimensional In Vitro Models

In contrast to simplified 2D models, 3D models more closely simulate the natural environment of cells and provide a more realistic representation of tissue interactions. Approaches include organoid and spheroid cultures, in which small three-dimensional cell aggregates mimic the architecture and function of tissue. Biopolymers and prefabricated scaffolds serve as physical supports for cell growth and tissue maturation, simulating the extracellular matrix. Hydrogels act as a three-dimensional scaffold for cells while creating a controllable microphysical environment. There are several different types of three-dimensional models. A distinction can be made between scaffolding-free models and models with scaffolding, as well as between static and dynamic, perfused models. [90].

To evaluate the impact of vitamin K_2_ on osteoporosis, Mandatori et al. established a co-culture system with human osteoblasts and osteoclasts derived from patients with primary osteoporosis that have been hospitalized after low-energy trauma. Both osteoblasts and monocytes were isolated from the same patient and were cultured together at a ratio of 2:1 (osteoblasts to monocytes) in a RCCS-4TM bioreactor (Synthecon™, Inc., Houston, TX, USA) at 4 rpm for 14 days to create bone-like constructs. After 24 h, they observed bone aggregates and treated them with vitamin K_2_, which resulted in an increase in osteoblastic activity and a decrease in osteoclast activity. Protein expression was evaluated using flow cytometry for alkaline phosphatase, Runx2, Collagen 1a1, and osteocalcin. Activity of osteoblasts was evaluated with alizarin red staining, while osteoclast activity was monitored by tartrate resistant acid phosphatase immunofluorescence staining. In addition, they evaluated gene expression of alkaline phosphatase, Runx2, osteopontin, collagen1a1, osteocalcin, and β2-microglobulin. Their results indicate that the in vitro 3-D experimental model reflects the patient’s bone metabolism and may be useful to predict personal responsiveness [99]. A similar approach was used to evaluate the effect of umbelliferon, a coumarin derivative, to evaluate its impact on bone mineralization (Pelusi et al.). They cultured peripheral blood mononuclear cells in a medium containing 50 ng/mL M-CSF and 30 ng/mL RANKL to differentiate the monocytes into osteoclasts. To achieve 3D bone-like models, they added 2 × 10^6^ osteoblasts and 1 × 10^6^ osteoclasts in an RCCS-4TM bioreactor (Synthecon™, Inc., Houston, TX, USA), starting at 4 rpm for two days. After two days, the bioreactor was set to 16 rpm. Read out parameters were: 3-(4, 5-Dimethylthiazolyl-2-yl)-2, 5-Diphenyltetrazolium Bromide (MTT) Cell Metabolic assay, alizarin red staining, real time PCR (ALP, COL1a1, OC, estrogen receptor 1, catenin 1, glyceraldehyde-3-phosphate dehydrogenase), flow cytometry (ALP, OPN, OC, COL1a1), immunofluorescence (expression of β-catenin) and immunocytochemistry (TRAP). Their results indicate that there is a potential effect of umbelliferon in bone health to prevent/treat bone loss diseases such as osteoporosis [100]. The group of Hulley et al. performed several different methods of inducing osteoclastogenesis and tested the potentially anti-resorptive drug FG-4592, a HIF stabilizing compound. They established co-cultures using commercially available osteoblasts and leukocytes to study osteoclast formation, comparing proliferating osteoblasts supplemented with M-CSF and RANKL, proliferating osteoblasts alone as a control, and differentiated osteoblasts to evaluate their ability to induce osteoclasts. In these co-culture setups, an anti-catabolic effect of the HIF stabilizing substance FG-4592 was observed [101]. In summary, 3D in vitro bone models more closely replicate the physiological environment by enabling complex cell–cell and cell–matrix interactions, making them powerful platforms to study disease mechanisms and to evaluate therapeutic compounds in osteoporosis research.

### 3.3. Ex Vivo Models/Explants

Explants refer to the cultivation of tissue or bone samples obtained ex vivo. They provide an immediate platform for the investigation of tissue and cell processes under near-physiological conditions. The use of transwell systems or culture plates is often used to enable the cultivation and examination of bone fragments and to gain a better understanding of osteoporosis pathology. However, it should be noted that bone grafts are often performed with less relevant or immature bone or with material of non-human origin that is not transferable to humans. [102].

In a study by Salamanna et al., they analyzed the connection between osteoporosis and breast cancer. They used human bone sections from healthy and osteoporotic patients and co-cultivated them with the breast cancer cell line, MCF-7. The evaluated parameters were bone tissue viability using alamar blue test, vascular-endothelial growth factor receptor 1 and 2, OPG, RANKL, interleukin 6 (IL-6), interleukin 1b (IL-1b), interleukin 10 (IL-10), and tumor necrosis factor alpha (TNF-a), VEGF, and interleukin 8 (IL-8) in cell culture supernatant using ELISAs and Milliplex^®^ assays. After culture, bones were sectioned and stained using haematoxylin/eosin (H&E). For immunohistochemistry, antibodies against keratin 8 and 18 were used. They observed significant differences in all evaluated parameters in the cell culture supernatants. Regarding osteoporosis, the changes in the OPG/RANKL ratio between the healthy and osteoporotic bone specimens may be highlighted [103]. Taken together, explant models allow near-physiological investigation of bone processes but are sometimes limited by immature or non-human material.

### 3.4. On-a-Chip Models

Organ-on-a-chip systems combine microfluidic technologies and cell culture techniques to precisely simulate physiological conditions and complex cell interactions. They enable the visualization of multi-cell interactions under controlled conditions and the simulation of mechanical forces, as they occur in bone tissue during movement or loading, to investigate the response of cells to mechanical signals. With these technological advances, on-a-chip in vitro models resemble biomimetic conditions closer to physiology. Furthermore, on-a-chip models offer the possibility of simulating pathological conditions [104].

Paek et al. used mouse osteocytes IDG-SW3 and osteoblasts MC3T3-E1 to form a three-dimensional construct of an osteon, the functional unit in cortical bone [105]. They established a hydrogel consisting of collagen type I and osteoblast-derived decellularized extracellular matrix. With this model, they used an anti-sclerostin antibody to test their AI-assisted image analysis software to evaluate the effects of anti-osteoporotic drugs [106]. Another interesting on-a-chip platform focuses on the setup of a platform to resemble bone marrow 3D in vitro. Since osteoporosis is a disease affecting the entire osseous system, this study might be a step towards a functional 3D in vitro model of complete bones. The group of Glaser et al. cultivated CD34^+^ hematopoietic stem cells and CD66b^+^ neutrophils in a co-cultivation setup [107]. Vis et al. use human bone marrow-derived MSCs, which they seeded on a chip and differentiated to osteoblasts. After 21 days, monocytes were added. To supplement the differentiation of monocytes to osteoclasts, RANKL and M-CSF were added. A self-assembling bone-like tissue was observed, which showed characteristic expression of osteopontin and collagen-1 as markers of osteoblast activity and activity of tartrate resistant acid phosphatase, as well as cross-linked C-telopeptide of collagen type 1 as an activity marker of osteoclasts [108]. Therefore, organ-on-a-chip models mimic physiological and mechanical conditions with high precision, enabling advanced studies of bone cell interactions and drug testing, demonstrating their potential for osteoporosis research.

### 3.5. In Silico Models

In silico models use computer-aided methods and simulations to investigate molecular mechanisms and develop new therapeutic approaches. Network analyses enable the investigation of biological networks to identify molecular targets and signaling pathways of osteoporosis. Target identification is used to discover potential therapeutic target structures. Molecular dynamics simulates interactions and movements at the molecular level, while ligand-protein docking models the binding of drugs to target molecules to predict the efficacy of potential therapies. Therefore, these in silico models do not require any biological material and instead are virtual mathematical simulations of biological systems capable of representing complex systems [109].

The study by Imanpour et al. aimed to find new antagonists for miRNAs and siRNAs that target genes associated with osteoporosis. They found 21 genes related to bone regeneration using the Regeneration Gene Database and 40 genes associated with osteoporosis using the Disgenet database. The interactions between the proteins of the genes identified were analyzed using GeneMANIA, which is a tool that predicts the potential functions of a gene. Concerning the study of miRNAs and siRNAs, several different bioinformatic tools were used, including Enrichr for the identification of miRNAs and TargetScan for their potential binding sites. Furthermore, they wanted to identify optimal nanocarriers to transport these RNAis in the body. To do this, they used CHARMM GUI to design polymeric structures that would be suitable as nanocarriers for transporting the interfering RNAs [110].

A study by Hassan et al. aimed to find novel targets for the treatment of osteoporosis by identifying the genetic causes of extremely high bone mass in a cohort from the United Kingdom high bone mass study and the Anglo-Australasian Osteoporosis Genetics Consortium. They excluded all findings of extremely high bone mass caused by artifact or known causes of high BMD. After performing whole exome sequencing, they identified the missense variant NM_004482.4:c.1657C  >  T, p.Arg553Trp in *GALNT3* gene in a pedigree of a family. Another individual in the cohort had the mutation NM_004482.4:c.831 T  >  A, p.Asp277Glu in the same gene. To find out the effects of these mutations, they used the crystal structure of T. guttata GalNac-T3 gene complex with uridine diphosphate, manganese, and FGF23 as a template for human GalNac-T3 (the product of the gene GALNT3). The model was built using SWISS-MODEL and mutations were introduced with PyMOL. In addition, a genome-wide association study was performed to identify other GALNT3 variants that have effects on the bone mineral density. Simulations of the mutations p.Arg553Trp and p.Asp277Glu showed that they do affect GALNT3 enzymatic function. As this protein is associated with FGF23, its receptors FGFR1 and the co-receptor klotho, they looked for associations with bone mass. Since no effect was observed, they concluded that mutations in GALNT3 might affect a pathway other than FGF23 associated with bone metabolism [111].

Another study by Huang et al., which aims to identify new potential targets for osteoporosis treatment, discovered a binding site on soluble RANKL (sRANKL) that can be targeted to inhibit soluble RANK-RANKL interactions. Mouse RANKL was simulated based on crystal structure from the protein data bank, using the CUDA version of PMEMD (Particle Mesh Ewald Molecular Dynamics). For identification of small molecules inhibitors of sRANKL, the Triangle Mathcer method was used, and hits were evaluated using the London dG scoring method. From a library of 10,016 compounds for stronger binding to sRANKL than mRANKL (membrane-bound RANKL), 51 candidate compounds were evaluated in in vitro assays like osteoclastogenesis assay and surface plasmon resonance, finding that S3-15 is the most potent inhibitor of sRANKL [112].

A study by Kato et al. described a case of early-onset osteoporosis in a 47-year-old male who had a heterozygous ENPP1 (c.536A > G, p.Asn179Ser) mutation. In silico testing predicted this mutation to be pathogenic with a catalytic loss of velocity of about 45% compared to the wild-type. The ectonucleotide pyrophosphatase/phosphodiesterase 1 (ENPP1) hydrolyzes adenosine triphosphate into pyrophosphate and adenosine monophosphate, and is associated with loss of phosphate and high levels of FGF23, leading to impaired bone structure [113].

Govindan et al. evaluated the impact of thyroid stimulating hormone (TSH) on osteoporosis. They evaluated the effect of CREB and ELK1, which are mediated by TSH signaling, and interact with Runx2, a key mediator of osteoblastic differentiation. For homology models of the proteins involved, the SWISS-MODEL web server was used, for the interaction of RUNX2, CREB, and ELK1, the Patch Dock technique was used [114]. To sum up, in silico models use computational simulations to study molecular mechanisms, identify therapeutic targets, and predict drug effects without biological material.

### 3.6. Ability to Resemble Osteoporosis

It remains the question whether these models can resemble osteoporosis in humans. The previously cited 2D-model mimicking smoking-induced osteoporosis exhibits characteristic elevation of the RANKL/OPG ratio, as it has already been shown in a clinical study of Azizieh et al. that this ratio could be a useful marker in the diagnosis of osteoporosis. Furthermore, treating these models with bisphosphonates can bring this ratio back to the level of the untreated control [93,115]. To our knowledge, the RANKL/OPG ratio is still not used in the routine laboratory for the diagnosis of osteoporosis in addition to the standard parameters of bone remodeling such as bone-specific alkaline phosphatase, osteocalcin, N-terminal pro-peptide of type I procollagen (PINP), N-telopeptide (NTX), C-telopeptide (CTX), and pyridinoline crosslinks [116].

In contrast to 2D models, 3D in vitro models are advantageous due to their controlled microenvironment and ability to mimicking the complex structure of the human bone [102]. A study by Pelusi et al., using cells from patients with osteoporosis, they were able to build a functional 3D model of osteoporosis and evaluate the individual responsiveness for test drugs, in this case, vitamin K_2_ [100].

The previously cited paper used an ex vivo cultured piece of osteoporotic bone to determine the relationship between bone loss and breast cancer. A great advantage of this approach is the possibility of assessing the unique bone microenvironment of a single patient [103]. A possible use of this methodology may be to use healthy bone samples and treat them with drugs that are suspected of inducing secondary osteoporosis. Otherwise, using patient bone samples may come with greater variability.

Organ-on-a-chip models allow the design of more complex microenvironments, e.g., vascularization of a bone model as shown before by Glaser et al., Paek et al., or Vis et al. [106,107,108]. To our knowledge, there are no on-a-chip platforms that mimic osteoporosis despite the many platforms that have been developed for testing potential anti-resorptive drugs. Using on-a-chip platforms might be a great advantage for providing a deeper understanding of the complex pathomechanisms involved in the emergence of osteoporosis.

### 3.7. Limitations

A common limitation to in vitro models is that they usually only simulate single organs in isolation from an organ system, unlike in vivo models. Most in vitro models contain only a few or a single cell type. The model designed by Salamanna et al. circumvents this issue by using whole bone samples. However, using an entire bone brings the change of having no standardized samples [103]. A possible solution for obtaining more standardized results is the use of cell lines, as was shown in Paek et al. [106].

As osteoporosis may have an inflammatory component, as Ginaldi et al. described earlier, the integration of immune cells in the established osteoporosis in vitro model might be a necessary next step.

Regarding in silico models, all models reviewed in this article focus on identifying new drugs or targets for the treatment of osteoporosis, and interactions of single proteins rather than studying mechanisms leading to osteoporosis. However, a major advantage of in silico models compared to in vitro or in vivo models is the ability to study many potential targets in a short time [117].

## 4. Conclusions

Animal models remain frequently used in the study of osteoporosis, with mice and rats representing the most common. The value of these models for generating knowledge and new approaches for the treatment of osteoporosis in humans cannot be denied. However, the clinical need for new developments to combat this widespread disease is still enormous. The development of the age pyramid, particularly in the western world, poses major challenges for research at present and especially in the future. Animal models in the field of osteoporosis are useful in basic science, as well as to generate approaches for the development of translational strategies. While gaining scientific knowledge is undoubtedly important, the future increasingly demands that human patients play a central role in research to adequately combat important diseases in the population. Recently, there has been an increase in the establishment of human in vitro models. These range from simple 2D cell cultures to highly sophisticated, specific three-dimensional models using human primary cells or tissues, using the latest devices such as on-a-chip systems. This offers both better proximity to the human system and transferability of the results compared to animal models. However, these model systems are limited by the absence of whole organ systems and a lack of cell type diversity, as they generally only consist of cells directly involved in the disease. An optimal model for osteoporosis, therefore, does not currently exist. Nevertheless, enormous progress has been made in the field of in vitro modeling in recent years. An optimal in vitro model should ideally consist of human cells that are evaluated by state-of-the-art technologies that are also clinically established (e.g., micro-Ct) and should be validated using clinically relevant parameters.

## Figures and Tables

**Table 1:** Triggers of secondary osteoporosis.

Trigger	Specification
Drugs	Alcohol, anticoagulants, anticonvulsants, antidepressants, aromatase inhibitors, medroxyprogesterone acetate, GnRH agonists, cyclosporines, calcineurin inhibitors, GCs, loop diuretics, proton pump inhibitors, thiazolidinediones
Gastrointestinal disorders	Celiac disease, inflammatory disease, gastric bypass, anorexia nervosa, hemochromatosis, chronic liver disease
Hematological disorders	Monoclonal gammopathy of uncertain significance, multiple myeloma, systemic mastocytosis, beta thalassemia major
Rheumatic diseases	Rheumatoid arthritis, sarcoidosis, systemic lupus erythematosus
Endocrine disorders	Acromegaly, cushing syndrome, diabetes mellitus, hypogonadism, thyroid dysregulation, growth hormone deficiency
Nutritional deficiencies	Calcium, magnesium, phosphate, vitamin D
Renal disorders	Idiopathic hypercalciuris, renal tubular acidosis, chronic kidney disease

**Table 2:** Anti-resorptive drugs for osteoporosis treatment [31,35].

Class of Drug	Examples
Estrogens	
Selective estrogen receptor modulators	Bazedoxifene, raloxifene
Bisphosphonates	Alendronic acid, risendronic acid, zolendronic acid, ibandronic acid
Antibodies	Denosumab, Romosozumab
PTH derivates	Teriparatide, abaloparatide

**Table 3:** Animal models for osteoporosis research [38,42,43,44].

Species	Purpose—Basic Research	Purpose—Translational Research	Similarity to Human Bone
Mouse	Understanding the (patho)physiology Getting mechanistical insights	Identification of new targets and therapeutic strategies	Macrostructure + Microstructure + Remodeling +
	Understanding cellular influences	Proof-of-concept for new compounds	
Rat	Studying the pathophysiology Focus on biomechanical cues	Proof-of-concept for new compounds Testing of new biomaterials	Macrostructure + Microstructure + Remodeling +
		Testing of new biomaterials	
Dog	Focus on remodeling processes	Preclinical drug testing Testing for osteosythesis	Macrostructure + Microstructure ++ Remodeling ++
Sheep/Goat	Rarely used	Testing of implants Testing of operation techniques Preclinical drug testing	Macrostructure ++ Microstructure + Remodeling ++
Non-human primates		Testing of implants Testing before humans	Macrostructure +++
			Microstructure +++ Remodeling +++

+ indicates a rather low similarity, ++ indicates a moderate similarity, and +++ indicates a high similarity to human bone.

**Table 4:** Models for postmenopausal osteoporosis.

Ovariectomy		Hormonal Manipulation		Naturally Aging	
Advantages	Drawbacks	Advantages	Drawbacks	Advantages	Drawbacks
- Well established - Diversified data	- Stress and strain - Educated surgeon	- Not invasive - Well controllable	- Chemically induced - Massive side effects	- Not invasive - No stress for animals	- Long timeframe - Low productivity

**Table 5:** Overview of models for disuse osteoporosis with advantages and disadvantages.

Tail Suspension		Immobilization		Transgenic Models	
Advantages	Drawbacks	Advantages	Drawbacks	Advantages	Drawbacks
- Well established - Diversified data	- Stress and pain - Effects on other physiological systems	- targeted examination of specific limbs - easy-to-use	- Stress and pain	- Specific tasks - No stress for animals	- non-specific effects

## Data Availability

No new data were created or analyzed in this study.
